# Classifying the metal dependence of uncharacterized nitrogenases

**DOI:** 10.3389/fmicb.2012.00419

**Published:** 2013-01-30

**Authors:** Shawn E. McGlynn, Eric S. Boyd, John W. Peters, Victoria J. Orphan

**Affiliations:** ^1^Division of Geological and Planetary Sciences, California Institute of TechnologyPasadena, CA, USA; ^2^Department of Chemistry and Biochemistry, the Astrobiology Biogeocatalysis Research Center, Montana State UniversityBozeman, MT, USA

**Keywords:** nitrogenase, metalloenzyme, molecular similarity, sequence conservation, vanadium, iron, molybdenum

## Abstract

Nitrogenase enzymes have evolved complex iron–sulfur (Fe–S) containing cofactors that most commonly contain molybdenum (MoFe, Nif) as a heterometal but also exist as vanadium (VFe, Vnf) and heterometal-independent (Fe-only, Anf) forms. All three varieties are capable of the reduction of dinitrogen (N_2_) to ammonia (NH_3_) but exhibit differences in catalytic rates and substrate specificity unique to metal type. Recently, N_2_ reduction activity was observed in archaeal methanotrophs and methanogens that encode for nitrogenase homologs which do not cluster phylogenetically with previously characterized nitrogenases. To gain insight into the metal cofactors of these uncharacterized nitrogenase homologs, predicted three-dimensional structures of the nitrogenase active site metal-cofactor binding subunits NifD, VnfD, and AnfD were generated and compared. Dendrograms based on structural similarity indicate nitrogenase homologs cluster based on heterometal content and that uncharacterized nitrogenase D homologs cluster with NifD, providing evidence that the structure of the enzyme has evolved in response to metal utilization. Characterization of the structural environment of the nitrogenase active site revealed amino acid variations that are unique to each class of nitrogenase as defined by heterometal cofactor content; uncharacterized nitrogenases contain amino acids near the active site most similar to NifD. Together, these results suggest that uncharacterized nitrogenase homologs present in numerous anaerobic methanogens, archaeal methanotrophs, and firmicutes bind FeMo-co in their active site, and add to growing evidence that diversification of metal utilization likely occurred in an anoxic habitat.

## INTRODUCTION

The majority of N in the biosphere is in the kinetically stable form of dinitrogen (N_2_). The only known biological means of accessing this vast reservoir of N_2_ is via the activity of nitrogenase, a complex metalloenzyme that catalyzes the reduction of N_2_ to ammonia (NH_3_). Nitrogenase comprises a two component enzyme whereby in an ATP-dependent process, electrons are shuttled by the nitrogenase iron protein (NifH) to the dinitrogenase reductase (NifDK) which harbors the active site (Bulen and LeComte, 1966).

**FIGURE 1:**
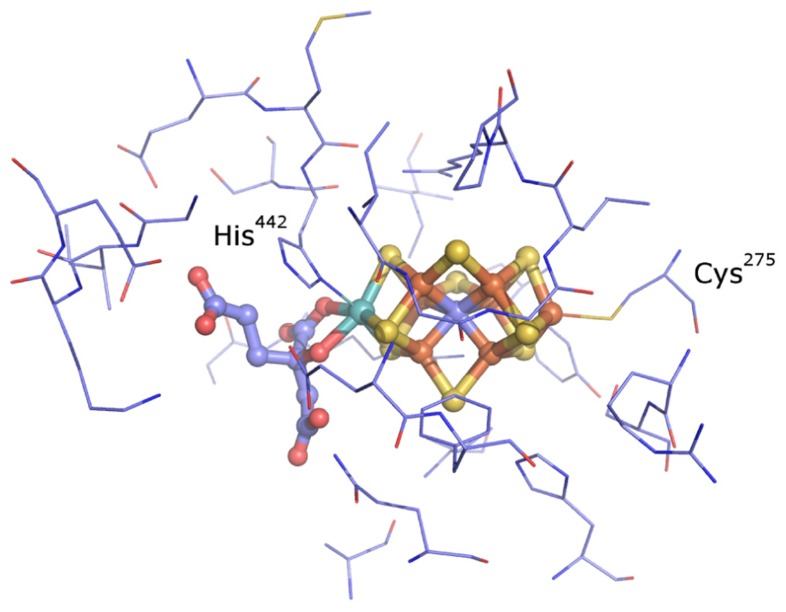
**The active site of nitrogenase from *A.vinelandii*** (Einsle et al., 2002; Lancaster et al., 2011; Spatzal et al., 2011). The metal cofactor–homocitrate complex is shown as balls and sticks and the residues found to be within 5 Å of the cofactor are shown as lines (thin). *Colors*: light blue, carbon; blue, nitrogen; red, oxygen; yellow, sulfur; rust, iron; cyan, molybdenum.

The nitrogenase active site consists of a biologically unique iron (Fe)–sulfur (S) cluster [X–7Fe–C-9S] where “X” is either molybdenum (Mo), vanadium (V), or iron (Fe) (**Figure 1**) (Einsle et al., 2002; Lancaster et al., 2011; Spatzal et al., 2011). These clusters are referred to by their metal type as FeMo-co, FeV-co, and FeFe-co, respectively. The cluster is ligated by non-protein homocitrate as well as cysteine and histidine side chains (Shah and Brill, 1977; Einsle et al., 2002). Biochemical studies have been conducted on representatives of Mo-dependent nitrogenase (Nif), V-dependent nitrogenase (Vnf), and Fe-dependent nitrogenase (Anf) (Robson et al., 1986; Eady et al., 1987; Chisnell et al., 1988; Eady, 1996). These different classes of nitrogenase have in common the ability to catalyze the reduction of N_2_ to produce NH_3_ with concomitant reduction of protons to molecular hydrogen (H_2_). However, they differ in their catalytic rates and efficiencies including the stoichiometry of NH_3_ and H_2_ produced per mol N_2_ reduced (Eady, 1996).

**FIGURE 2:**
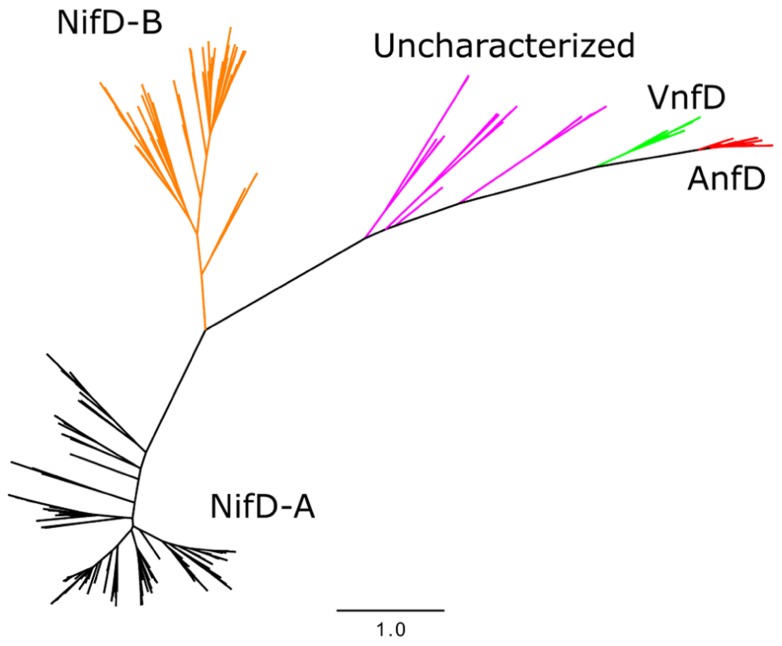
**Maximum likelihood unrooted phylogram of nitrogenase sequences**. FeMo-co NifD-A and NifD-B sequences in black and orange, respectively, uncharacterized nitrogenases in purple, AnfD in red, and VnfD in green.

Phylogenetic analyses of nitrogenase amino acid sequences derived from available genome databases indicate that nitrogenase homologs cluster into five major lineages (Raymond et al., 2004; Glazer and Kechris, 2009; Boyd et al., 2011a,b; **Figure 2**). These lineages comprise two Nif nitrogenase groups, as well as the Vnf and Anf groups. Between the Nif and Anf lineages is a separate lineage comprised of biochemically uncharacterized nitrogenases present in the genomes of representatives of the hydrogenotrophic methanogens, methanotrophic archaea, and firmicutes. Members of this lineage appear to be functionally competent as organisms which encode homologs are capable of N_2_ fixation (Mehta and Baross, 2006; Dekas et al., 2009). Previously, these homologs have been referred to as “uncharacterized nitrogenase” and were shown to form a lineage that branches later than Nif, Vnf, and possibly Anf, indicating that they evolved after metal differentiation in the active site cluster occurred (Boyd et al., 2011b). This observation has prompted questions regarding the metal composition and reactivity of the active site cluster present in these nitrogenase homologs (Boyd et al., 2011b).

In addition to the influence that active site metal composition has on reactivity, studies have shown that the peptide environment surrounding the active site cofactor also influences the enzyme activity of nitrogenase. For example, substitutions of amino acid residues in close proximity to the FeMo-cofactor (FeMo-co) N_2_ binding site of nitrogenase resulted in a Nif^-^ phenotype in *A. vinelandii* and altered the electronic and substrate reduction properties of the protein (Scott et al., 1990). Other residues which surround the active site have also been shown to dramatically alter substrate range of the enzyme (Mayer et al., 2002; Benton et al., 2003; Seefeldt et al., 2009). These experimental results have been instrumental in implicating individual roles for various amino acid residues surrounding the nitrogenase active site in proton transfer, local movement of nearby side chains, substrate binding, reaction intermediate stabilization, and/or modulating the electronic state of the cluster. Thus, both the metal composition of the active site cofactor and the composition of residues lining the active site cavity influence the reactivity of nitrogenase.

In this study, phylogenetic reconstruction and structural modeling were used to compare nitrogenase homologs, specifically to gain insight into the composition of the cluster at the active site of the biochemically uncharacterized nitrogenases. Active site volumes, inferred from predicted structures, and the amino acid environment that surrounds the metallocofactor were compared among nitrogenase homologs. In addition, models and known crystal structures of nitrogenase were compared by structural relatedness. These data provide new insights into the metal cofactor binding proclivity of nitrogenase enzymes and extend our understanding of the primary sequence and structural variation of nitrogenase. Additionally, this work adds further support to the concept that the first nitrogenase was likely to contain a Mo-based active site cofactor (Boyd et al., 2011a,b).

## MATERIALS AND METHODS

### SEQUENCE AND PHYLOGENETIC ANALYSIS

NifD homologs were obtained from the NCBI non-redundant database through BLASTp searches using NifD, VnfD, and AnfD proteins from *A. vinelandii* as queries (YP_002797379, YP_002797497, and YP_002801974, respectively). In the case of NifD searches, sequences were compiled using Pattern Hit Initiated BLAST (PHI-BLAST) specifying the NifD conserved “CXRS” amino acid pattern, where X is variable. In addition, a length criterion of 300–700 amino acids was imposed by the [slen] command.

Sequences were aligned using the EMBL ClustalW2 server with default parameters (Gonnet weight matrix) and manipulated with Jalview (Clamp et al., 2004; Waterhouse et al., 2009) and ClustalX (Larkin et al., 2007). Sequences were examined for known catalytic residues as reported in Boyd et al. (2011a). Phylogenies were generated using the PhyML webserver (Guindon et al., 2005) and calculated using the maximum likelihood method (Guindon and Gascuel, 2003; Guindon et al., 2010) using Shimodaira–Hasegawa-like aLRT supports and the LG substitution matrix (Anisimova and Gascuel, 2006). The resulting phylogram was projected using Fig Tree version 1.3.1^1^, and naming accomplished using the REFGEN/TREENAMER online web server (Leonard et al., 2009). Sequence alignments for each of the protein classes used in this study are available upon request from the authors. Analysis of amino acid conservation was judged by the presence of a * (conserved) or a colon above the alignment in ClustalW corresponding to amino groups of strongly similar properties (scoring >0.5 in the Gonnet PAM 250 matrix).

### STRUCTURE PREDICTION

Models of NifD and NifD homologs were generated through sequence submission to the iterative threading assembly refinement (I-TASSER) server (Zhang, 2008, 2009; Roy et al., 2010). The top model based on C-score (Wu et al., 2007) was selected for further analysis. The C-scores are derived from calculating convergence of intermediate structures that are produced during the I-TASSER run and range from -5 to 2 with high scores signifying models with high confidence (Zhang, 2008). Structures were visualized using PyMol^2^. Models were selected in an effort to obtain a representative diversity from each nitrogenase lineage. In the case of uncharacterized nitrogenases, these included nitrogenase associated with the archaeal methanogen* Methanocaldococcus infernus* ME (YP_003615674.1), an uncultured archaeal methanotroph believed to belong to the ANME-2c clade (ADF27322.1; Pernthaler et al., 2008), and the firmicutes *Candidatus* Desulforudis audaxviator MP104C (YP_001716346.1) and *Syntrophothermus lipocalidus* DSM 12680 (YP_003703435.1). A NifD homolog from a ANME-1 phylotype (Meyerdierks et al., 2010) was not included in this analysis as the sequence lacks ligand binding residues and is likely a “Nif-like” protein which are conserved in methanogenic archaea (Staples et al., 2007).

### STRUCTURAL COMPARISONS

Inferred structures were compared using an “all against all” comparison performed by the ProCKSI server. The relatedness of structures was calculated with standardized distance matrix derived from the Vorolign V-score (Birzele et al., 2007) and hierarchical clustering accomplished using the Complete Link (furthest neighbor) method (Defays, 1977) with the Clustering Calculator server^3^.

Active site volumetric calculations were performed on the CASTp server with a probe radius of 1.4 Å (Dundas et al., 2006). Cavities were manually inspected in each case to ensure correspondence with the nitrogenase metal cofactor-binding pocket. In order to identify putative conserved active site second shell residues, representative nitrogenase structures from the Protein Data Bank (PDB; Berman et al., 2003) and those created through homology modeling were structurally aligned with the Pymol program. The amino acids within 5 Å of the metal cofactor in the *A. vinelandii* 1M1N structure (Einsle et al., 2002), including homocitrate, were selected for analysis.

## RESULTS

### NITROGENASE HOMOLOG PHYLOGENY

Phylogenetic reconstruction of representative AnfD, VnfD, NifD, and uncharacterized D homologs revealed patterns of clustering that correspond to metal utilization (**Figure 2**), a finding that is consistent with phylograms reported previously (Raymond et al., 2004; Boyd et al., 2011a,b; Dos Santos et al., 2012). Two lineages comprising Nif were identified, denoted here as NifD-A and NifD-B. The uncharacterized homologs, in relation to Nif, do not resolve into a monophyletic lineage indicating that their phylogenetic placement based solely on D protein divergence should be interpreted with caution. However, the overall branching order of Nif and uncharacterized nitrogenase, whereby VnfD branch basal to AnfD, is consistent with the branching order observed for concatenations of HDK homologs reported previously (Boyd et al., 2011b).

### GLOBAL STRUCTURAL COMPARISON

**FIGURE 3:**
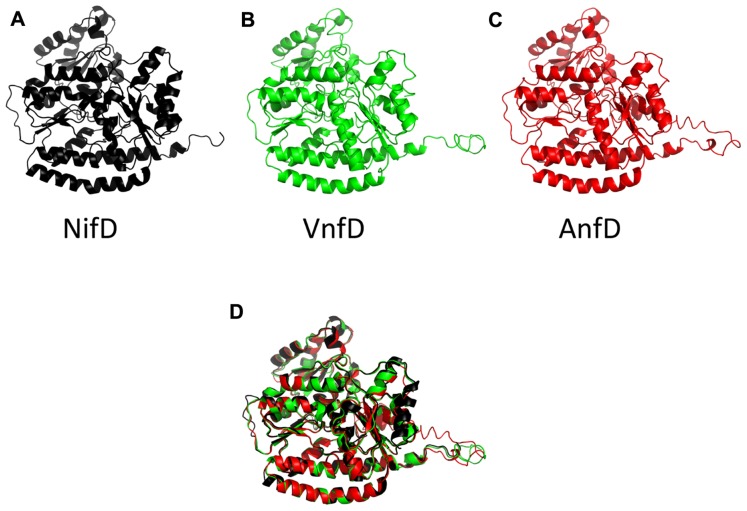
**Cartoon diagram of the *A.vinelandii* nitrogenase D subunits**. The NifD structure, drawn from 1M1N is in black **(A)**, and predicted structures for the *A. vinelandii* vanadium (VnfD) and Fe-only (AnfD) homologs are in green **(B)** and red **(C)**, respectively. A structural superposition of the three as performed in PyMol is shown in **(D)**.

Inferred nitrogenase homolog structures displayed modeling scores of high confidence (C-score, **Table 1**). These scores range from [-5,2] and models with C-score >-1.5 generally have a correct fold (Roy et al., 2010). In addition, root mean square deviation (RMSD) values obtained through comparison using the program DALI-LITE (Holm and Park, 2000) indicated that the models match very closely to the alpha chain of the high resolution NifD structure 1M1N (Einsle et al., 2002). Similarity can be visually depicted by an overlay of all *A. vinelandii *inferred structures (**Figure 3**).

**Table 1:** Protein structure modeling statistics.

Organism	Accession	Metal center	Type	C-score^§^	TM-score^§^	RMSD^⌘^
*Geobacter metallireducens *GS-15	YP_383630.1	FeMo-co	NifD-A	1.53	0.93 ± 0.06	0.7
*Desulfitobacterium hafniense* Y51	YP_520503.1	FeMo-co	NifD-A	1.00	0.85 ± 0.08	0.7
Uncultured archaeon (ANME-2)	ADF27322.1	FeMo-co^#^	Unc.	1.91	0.99 ± 0.04	0.7
*Ca*. Desulforudis audaxviator MP104C	YP_001716346.1	FeMo-co^#^	Unc.	1.20	0.88 ± 0.07	0.9
*Syntrophothermus lipocalidus* DSM 12680	YP_003703435.1	FeMo-co^#^	Unc.	1.26	0.89 ± 0.07	0.6
*Methanocaldococcus infernus *ME	YP_003615674.1	FeMo-co^#^	Unc.	0.72	0.81 ± 0.09	0.9
*Clostridium kluyveri *DSM 555	YP_001396457.1	FeMo-co	NifD-B	1.59	0.94 ± 0.05	1.6
*Methanosarcina acetivorans* C2A	NP_618769.1	FeMo-co	NifD-B	1.54	0.93 ± 0.06	1.4
*Methanosarcina mazei* Go1	NP_632746.1	FeMo-co	NifD-B	1.69	0.95 ± 0.05	1.4
*Azotobacter vinelandii* DJ	P16266.3	FeFe-co	AnfD	-0.37	0.67 ± 0.13	0.8
*Clostridium kluyveri* DSM 555	YP_001393772.1	FeFe-co	AnfD	-0.04	0.71 ± 0.12	0.9
*Methanosarcina acetivorans* C2A	NP_616149.1	FeFe-co	AnfD	-0.40	0.66 ± 0.13	0.9
*Rhodobacter capsulatus*	Q07933.1	FeFe-co	AnfD	-0.59	0.64 ± 0.13	0.9
*Azotobacter vinelandii* DJ	YP_002797497.1	VFe-co	VnfD	1.66	0.95 ± 0.05	0.8
*Clostridium kluyveri* DSM 555	YP_001395137.1	VFe-co	VnfD	1.76	0.96 ± 0.05	0.8
*Methanosarcina acetivorans* C2A	NP_616155.1	VFe-co	VnfD	0.67	0.80 ± 0.09	0.9
*Rhodopseudomonas palustris* CGA009	NP_946728.1	VFe-co	VnfD	1.64	0.94 ± 0.05	0.8

*C score and TM-scores are internal I-TASSER generated scores for estimating the quality of predicted models. C-scores exist in a range of [-5,2], where a C-score of higher value signifies a model with a high confidence (Wu et al., 2007; Roy et al., 2010). TM-score indicates topological similarity of protein structure pairs and estimated accuracy with a range of [0, 1]. A score of 1 indicates perfect structural similarity (Sali and Blundell, 1993). RMSD values in the table were calculated using DaliLite (Holm and Park, 2000) against the NifD subunit of the high resolution Nif 1M1N structure (Einsle et al., 2002). Uncharacterized nitrogenases are abbreviated as “Unc.”*

§ Internal I-TASSER generated score.

⌘ DaliLite calculated RMSD of model against PDB structure 1M1N.

# Cofactor content inferred in this work.

Among the obtained models, the AnfD structures display the lowest I-TASSER (least confident) modeling scores (**Table 1**). The AnfD models were examined by superimposing structures and manually inspecting them for structural differences. This led to the identification of a short C-terminal stretch of amino acids universally present in AnfD homologs, which is absent from other nitrogenase homologs. AnfD sequences in which this segment had been removed *in silico* prior to submission for homology model construction displayed markedly improved scores (C-score of 1.46 and 1.40 for *A. vinelandii* and *C. kluyveri* sequences, respectively), indicating that this section of the protein is responsible for lower model fit statistics (**Table 1**).

**FIGURE 4:**
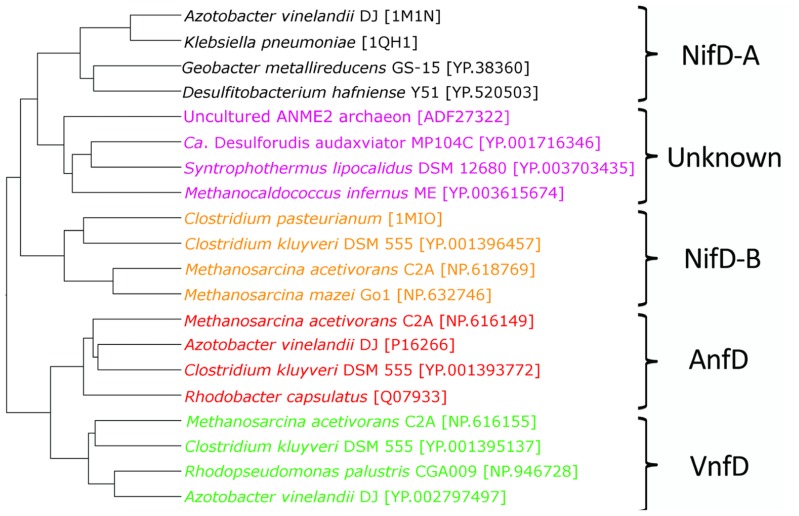
**Structural relationships among nitrogenase D subunits as calculated by Voronoi contacts and the complete linkage clustering method**. NifD-A are denoted in black, NifD-B in orange, uncharacterized nitrogenases in purple, AnfD in red, and VnfD in green.

Together with NifD structures obtained from the PDB [1M1N (Einsle et al., 2002), 1MIO (Kim et al., 1993), and 1QH1 (Mayer et al., 1999)], the inferred structures of AnfD, VnfD, NifD, and uncharacterized nitrogenase homologs were compared to examine whether D homologs can be resolved from other homologs at the structural level. The Vorolign V-score, which bases similarity on the conservation and evolutionary relationship of amino acid contact sets (i.e., Voronoi contacts) between proteins, resolved the various nitrogenase types into clusters by metal type without exception (**Figure 4**). AnfD and VnfD were resolved from NifD and uncharacterized D proteins. Uncharacterized D proteins cluster between two clades of NifD, a finding that is consistent with previous phylogenetic analyses of these proteins (Boyd et al., 2011b). This observation supports the hypothesis that the uncharacterized D protein homologs are structurally most similar to that of NifD and may harbor a similar cofactor to that present in the NifD active site (i.e., FeMo-co). In addition, these observations indicate that NifD, VnfD, and AnfD can be separated on the basis of inferred structure alone in a way that is largely consistent with the branching order based on phylogenetic reconstruction. The placement of the uncharacterized D homologs between the two NifD clades is different, however, between the sequence and structural clustering methods (purple groups, **Figures 2 and 4**). The discrepancy between structural and phylogenetic clustering is possibly the result of the unique structural features of the NifD-B group, which harbor an internal amino acid extension that all other types lack making them structurally distinct (Kim et al., 1993).

### ACTIVE SITE STRUCTURAL RELATIONSHIPS

A comparison of amino acids within 5 Å of the active site FeMo-co in inferred structures indicated that they comprise nine separate sequence areas or motifs (**Figure 1**; **Table 2**). For simplicity, the numbering scheme from that of *A. vinelandii* NifD was adopted when discussing these motifs. Two of these areas correspond to the two active site cofactor ligands Cys^275^ and His^442^ and have been discussed elsewhere (Glazer and Kechris, 2009). The remaining sequence areas analyzed here do not share bonds to the active site and can be considered to be a part of the second shell of amino acids which surround the cofactor. The amino acid distribution at these positions between the nitrogenase classes are listed in **Table 2**. A number of amino acids in these regions were conserved across all nitrogenase proteins (**Table 2**, bold-faced entries). Unique to each class however, the different nitrogenase types exhibit distinct patterns of conservation. For example, the conserved His^383^ residue is flanked by either a Glu or Gln residue at position 380 in NifD, whereas this position is occupied by the positively charged Lys^380^ in the VnfD and AnfD. Uncharacterized nitrogenase homologs display Thr, Leu, or Met, but never a positively charged residue in this position. Another notable substitution occurs near conserved Gly^424^, where Lys or Arg is present at position 428 in NifD and uncharacterized nitrogenases but is present as Gly in the V- and Fe-dependent nitrogenase.

**Table 2:** Sequence conservation near the nitrogenase active site.

Type	Sequence feature							
	G^66^	R^96^	Q^191^	N^230^	G^356^	H^383^	G^424^
NifD-A	**C**xyA**G**skG**V**VwG^^^	SxxxRRN*	**G**vx**QS**L**GH**H1a	dy**N**IGGd^&^	v**G**GLRxRH^§^	GYE**F**x**H**^€^	s**G**iKEKf
Unc.	**C**tyA**G**xxG**V**vxG^#^	R	**G**xs**QS**x**GH**h	iGdY**N**ixxD	ixx**G**xPrxWH 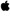	**F**x**H**^¶^	ix**G**xKEkfl
NifD-B	**C**xyA**G**xkG**V**v	sWxxRRn	**G**Vt**QS**x**GH**HiA	lGEY**N**IGGD	t**G**GSRxHxY	Gye**F**x**H**R	t**G**ikxky
AnfD	**C**AyC**G**AKH**V**IG^$^	TWqTKRYI	**G**PS**QS**G**GH**HKI	vGeY**N**IQGD^⌘^	lWx**G**GSKLWHW	vYtK**F**G**H**Q	ifT**G**xRPGE
VnfD	**C**afC**G**aKL**V**IGG	TWHTKRYP	**G**VS**QS**K**GH**Hxl	IGDf**N**IQGD	IWT**G**GPRLWHW	MSSK**F**G**H**q	ifT**G**PRVGxL
	62	92	188	226	353	377	421

*Sequence features are identified by a residue with a raised numeral indicating the *A. vinelandii *sequence number of the residue directly below it. Underlined residues are those within 5Å of the *A. vinelandii *active site cofactor. Residues in bold are conserved among all nitrogenases. Residue positions of lower case exhibit conservation of strongly similar amino acids. Areas with no conservation are indicated by an "x" or a blank space, and raised symbols note positions where an exception exists in the presence of an otherwise conserved residue. Sequence areas within three residues of the underlined positions are shown where conservation exists. Uncharacterized nitrogenases are abbreviated as “Unc.”*

^ C is G in *Cylindrospermum stagnale* AAN63669.1.

* S is R and second R replaced with C in *Paenibacillus fujiensis* BAH24166.1.

& Sequence conservation limited to “NIG” when including *Delftia tsuruhatensis *AAS55952.1.

§ First G position is replaced by A in *Frankia *sp. AAB36877.1.

€ *Lyngbya majuscula* CCAP 1446 AAY78884.1 contains the insertion “FA” after x.

# C is undetermined in Candidatus Desulforudis audaxviator YP_001716346.1.

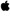
 G replaced with S in unnamed *Archaea* BAF96801.1.

¶ H is R in BAF96801.1.

$ First G is V in *Clostridium hungatei* AAB02935.1.

⌘ GxxxxxG if including *Clostridium hungatei* AAB02935.1.

Other differences in sequence conservation in D homologs include Gly^69^ and Val^70^. In biochemical assays, Gly^69^ substitution has been shown to result in resistance to acetylene inhibition, while Val^70^ substitutions confer the *A. vinelandii* Mo-dependent nitrogenase the ability to reduce short chain alkynes thereby increasing the substrate size range of the enzyme (Christiansen et al., 2000; Mayer et al., 2002; Benton et al., 2003; Seefeldt et al., 2009). Both Gly^69^ and Val^70^ are conserved in the Mo-dependent nitrogenase. However, His or Leu is observed at position 69 in AnfD and VnfD, respectively. Importantly, Gly^69^ is conserved in the uncharacterized nitrogenase homologs, adding further support to the hypothesis that uncharacterized nitrogenases bind FeMo-co.

To further compare the relatedness of nitrogenase homologs and investigate the potential for uncharacterized nitrogenases to bind FeMo-co, active site volume calculations were performed (Dundas et al., 2006). These calculations were motivated by the untested hypothesis that some of the observed catalytic differences between the nitrogenase types could be related to the nature of the FeMo-co binding cavity. Calculations were performed on three members of each respective lineage, in the absence of the active site cluster. Representatives included homology models generated during this study as well as structures obtained from the PDB (1M1N and 1MIO). The results indicate that calculated active site volumes varied markedly depending on active site cluster composition, with NifD having active site cavities of approximately double the size of those associated with VnfD and AnfD (**Table 3**). Likewise, the uncharacterized nitrogenase D homologs were shown to have an active site volume that was similar to NifD. These trends were observed both when the inferred structures were compared to each other and when inferred structures were compared with those obtained through x-ray crystallography (e.g., NifD; Kim et al., 1995; Mayer et al., 1999; Einsle et al., 2002). The similarity in uncharacterized nitrogenase active site volume to that of NifD, and the large differences in active site volume between uncharacterized nitrogenase D homologs and AnfD/VnfD support the hypothesis that uncharacterized nitrogenases most likely harbor an active site cofactor that is most similar to that present in Mo-dependent nitrogenase (i.e., FeMo-co).

**Table 3:** Average active site cofactor volume (*n* = 3 in each case) for nitrogenases computed by the CASTp server in the absence of the active site cofactor.

Nitrogenase type	Active site volume (Å^3^)	Average deviation
NifD-A	1701	178
Uncharacterized	1842	316
NifD-B	1931	113
AnfD	1198	351
VnfD	1015	168

## DISCUSSION

The diversity of reactions catalyzed by metalloenzymes is a consequence of cofactor composition and the protein environment in which these cofactors are bound. This study applies computational tools to examine the relationship between active site cofactor composition, primary sequence variation, and three-dimensional structure in nitrogenase homologs. The results of the analyses of nitrogenase homologs collectively suggest that the uncharacterized nitrogenases are most similar to Mo-nitrogenase and bind an active site metal center similar to FeMo-co. This conclusion is supported by (i) global structure similarity, (ii) comparison of calculated active site volume, and (iii) active site neighboring amino acid composition.

Further support for this conclusion can be derived from genomic comparison. In the genomes of completely sequenced organisms harboring uncharacterized NifD analyzed here (*n* = 10), AnfD and VnfD homologs are not found, consistent with a previous finding that alternative nitrogenases (Anf/Vnf) are only found in genomes that have a full complement of Nif genes (i.e., Anf and Vnf encoding genomes appear to always encode Nif; Boyd et al., 2011a). In contrast uncharacterized nitrogenase genes occur as the only nitrogenase homolog in their respective genomes. Furthermore these genomes lack genes which encode a third structural subunit (*anfG*, *vnfG*) found to be associated with known alternative nitrogenases (Chatterjee et al., 1997; Lee et al., 2009), making it unlikely that they bind either FeV-co or FeFe-co.

Mutagenesis studies have shown that the substrate reduction properties of nitrogenase are modified by amino acid substitutions near the active site (for examples, see Scott et al., 1990; Fay et al., 2007; Seefeldt et al., 2009; Peters et al., 2011). In addition, there are significant differences in the specific activities and substrate reduction properties observed for different metal types of nitrogenase. For example, V- and Fe-only nitrogenases have lower specific activities and divert a higher proportion of reducing equivalent to hydrogen production than Mo-nitrogenase (Bulen and LeComte, 1966; Robson et al., 1986; Eady, 1996; Schneider et al., 1997). These observations indicate that nitrogenase activity is significantly impacted by both the local peptide environment and metal composition.

Many of the amino acids surrounding the cofactor listed in **Table 2** are conserved between nitrogenase types, suggesting that these have a fundamental role in nitrogenase and were fixed early in the evolutionary history of nitrogenase. The majority of these residues are involved in direct coordination of nitrogenase metal clusters (P-cluster or FeMo-co in Mo-nitrogenase) or have been implicated in having some functional role through amino acid substitution studies (Scott et al., 1990; Benton et al., 2003; Barney et al., 2004; Igarashi et al., 2004; Lee et al., 2004; Seefeldt et al., 2009; Sarma et al., 2010; Yang et al., 2011). Other residues surrounding the cofactor that are conserved but only specifically within each form of nitrogenase are particularly diagnostic in terms of categorizing the uncharacterized nitrogenases. For example, the position of Arg^96^ (amino acid sequence of *A. vinelandii*) is conserved in NifD, whereas both the AnfD and VnfD have Lys in this position. The presence of Arg at this position in unclassified nitrogenase D homologs is suggestive of FeMo-co binding. The presence of a negatively charged glutamate at position 380 in NifD and a positively charged lysine in VnfD/AnfD is intriguing, as this position is located near the variable metal binding site of the nitrogenase cofactor. While uncharacterized nitrogenase D homologs do not exhibit conservation at this position, the amino acid observed at this position is not positively charged as it is in VnfD/AnfD. Other residues that may possibly delineate metal cofactor binding among nitrogenase homologs include position 65 in NifD. Here, both Mo-type and uncharacterized nitrogenase have a conserved Ala whereas both VnfD and AnfD have Cys. Near position 65, Gly^69^ of Nif and the uncharacterized lineage differs from the conserved His or Leu in Anf and Vnf, respectively. These observations are in line with the observation that in the environment of Cys^275^, which is a ligand of the nitrogenase cofactor, Mo-type nitrogenase exhibit the amino acid pattern Cys-Tyr-Arg-Ser, Cys-Gln-Arg-Ser, or Cys-His-Arg-Ser, whereas the V and Fe-only forms contain the sequence Cys-Ala-Arg-Ser (Glazer and Kechris, 2009). Together, these patterns suggest that the uncharacterized Nif bind FeMo-co.

In summary, the results presented here suggest that uncharacterized nitrogenase homologs present in the genomes of anaerobic methanogens, anaerobic methanotrophic archaea, and anaerobic firmicutes are likely to harbor FeMo-co. Previous phylogenetic studies indicate that the ancestral nitrogenase contained FeMo-co, and that a FeMo-co containing ancestor then diversified to give rise to alternative nitrogenase and uncharacterized nitrogenase homologs. That the uncharacterized nitrogenase homologs, which have yet to be identified in the genomes of aerobes or facultative anaerobes, harbor FeMo-co supports the notion that diversification of nitrogenase toward the use of alternative metals (e.g., V and Fe) likely occurred in an anoxic environment (Boyd et al., 2011b). Collectively, these observations contrast with hypotheses put forth based on the bioavailability of metals in marine environments and the evolution of nitrogenase, whereby Anf/Vnf were suggested to predate Nif (Anbar and Knoll, 2002; Raymond et al., 2004). Reconciling these observations, we suggest an important role for microenvironments and transient fluctuations in metal availability in driving the diversification of nitrogenase early during its evolutionary history.

## Conflict of Interest Statement

The authors declare that the research was conducted in the absence of any commercial or financial relationships that could be construed as a potential conflict of interest.

## Acknowledgments

Shawn E. McGlynn is an Agouron Geobiology Option postdoctoral fellow in the Division of Geological and Planetary Sciences at Caltech and is grateful for support provided by the Agouron Institute. Additional support for this work was provided by the Department of Energy Grant DE-SC0004949 (to Victoria J Orphan) and the NASA Astrobiology Institute (NAI) NNA08C-N85A (to John W. Peters and Eric S. Boyd). Eric S. Boyd also wishes to acknowledge support from the National Science Foundation (EAR-1123689 and PIRE-0968421). The authors are grateful for comments from Aaron D. Goldman, Joshua A. Steele, Wolfgang Nitschke, and members of the laboratory of Victoria Orphan.

## Footnotes

1 http://tree.-bio.ed.ac.uk/

2 http://www.pymol.org

3 http://www.biology.ualberta.ca/jbrzusto/cluster.php
